# A Viable Hypomorphic Allele of the Essential *IMP3* Gene Reveals Novel Protein Functions in *Saccharomyces cerevisiae*

**DOI:** 10.1371/journal.pone.0019500

**Published:** 2011-04-29

**Authors:** Bruno Cosnier, Marta Kwapisz, Isabelle Hatin, Olivier Namy, Sylvie Hermann-Le Denmat, Antonin Morillon, Jean-Pierre Rousset, Céline Fabret

**Affiliations:** 1 IGM, CNRS, UMR 8621, Orsay, France; 2 Université Paris-Sud, Orsay, France; 3 Université Pierre et Marie Curie, Paris, France; 4 Institut Curie, UMR 3244, Paris, France; 5 Ecole Normale Supérieure, Paris, France; University of Louisville, United States of America

## Abstract

In *Saccharomyces cerevisiae*, the essential *IMP3* gene encodes a component of the SSU processome, a large ribonucleoprotein complex required for processing of small ribosomal subunit RNA precursors. Mutation of the *IMP3* termination codon to a sense codon resulted in a viable mutant allele producing a C-terminal elongated form of the Imp3 protein. A strain expressing the mutant allele displayed ribosome biogenesis defects equivalent to *IMP3* depletion. This hypomorphic allele represented a unique opportunity to investigate and better understand the Imp3p functions. We demonstrated that the +1 frameshifting was increased in the mutant strain. Further characterizations revealed involvement of the Imp3 protein in DNA repair and telomere length control, pointing to a functional relationship between both pathways and ribosome biogenesis.

## Introduction

In *Saccharomyces cerevisiae*, the essential *IMP3* gene encodes a 183 amino acid component of the small ribosomal subunit (SSU) processome, which is required for pre-18S rRNA processing. This large ribonucleoprotein complex is composed of the nascent 35S pre-rRNA, the small nucleolar RNA U3 and of about 50 proteins. It was proposed that it assembled from a number of stable subcomplexes including the UTP-A/t-UTP subcomplex (Utp5p, Utp4p, Nan1p, Utp8p, Utp9p, Utp10p, and Utp15p), the Pwp2p/UTP-B subcomplex (Utp6p, Pwp2p, Utp18p, Utp21p, Utp13p, and Dip2p) which interacts directly with the 5′-ETS (external transcribed spacer) of the 35S pre-rRNA, the UTP-C subcomplex (Rrp7p, Utp22p, Ckb1p, Cka1p, Ckb2p, and Cka2p), and the Mpp10 subcomplex (Mpp10p, Imp3p, and Imp4p) [1], [2]. The Imp3 protein interacts with Mpp10p (Imp stands for “interacting with Mpp10p”) and mediates interactions of Imp4p and Mpp10p with the U3 snoRNA [3], [4]. The Imp3 and Imp4 proteins apparently increase the stability of the otherwise unstable U3 – ETS RNA hybrid [5], [6]. Yeast and human Imp3 proteins display ∼50% identity and 65% similarity, and their role in pre-rRNA processing is evolutionary conserved [7].

The yeast small 40S and large 60S ribosomal subunits are composed of the 18S rRNA and 33 ribosomal proteins and of the 25S, 5S, 5.8S rRNA and 48 ribosomal proteins, respectively. The early stages of ribosome assembly occur in conjunction with processing of the 35S pre-ribosomal RNA transcript [8], [9], [10], the first three cleavages being essential for production of the mature 18S rRNA and thus 40S small ribosomal subunit, but not for the 60S large ribosomal subunit [3], [11]. They occur in the large U3-associated ribonucleoprotein complex and require base pairing of the U3 snoRNA with the 5′-ETS and 18S rRNA [12], [13]. The released 20S, exported to the cytoplasm, and the 27SA_2_ precursors are, respectively, matured into 18S rRNA, and 25S plus two forms of 5.8S rRNA.

Depletion of any member of the t-UTP subcomplex results in decreased transcription of rDNA leading to decreased levels of the primary 35S rRNA transcript [14]. In contrast, mutation or depletion of most other members of the SSU processome complexes causes decreased 18S rRNA levels without affecting the levels of the 25S or 5.8S rRNA. More particularly, the depletion of either Imp3p, Imp4p, Mpp10p or of U3 snoRNA leads to an important decrease in 18S rRNA production, an accumulation of the aberrant 23S precursor, and reciprocally to a 20S precursor decrease and a 35S pre-rRNA accumulation [15].

Recently, an additional role of the U3 snoRNP was postulated in telomere function through the interaction between Imp4p and Cdc13p, a single-stranded telomere-binding protein involved in telomere maintenance [16]. The Imp4 protein was shown to specifically bind single-stranded telomeric DNA and to associate with telomeres *in vivo* [17]. Whether the Imp3 and Mpp10 proteins are also required for tight binding of Imp4p to telomeres is yet unclear.

In the present work, we report the analysis of a viable mutant of the *IMP3* gene. Mutation of the termination codon resulted in production of a C-terminal elongated protein. Studies revealed phenotypes similar to Imp3 protein depletion, but also demonstrated that the mutant protein induces an increase of the +1 frameshifting, a defect in double-stranded break repair, and a lengthening of telomere ends. Results point, for the first time, to a role of the Imp3 protein in pathways beyond ribosome biogenesis. The availability of a viable mutant allele of *IMP3* is likely to be of significant importance in elucidating functions of the protein.

## Results

### Generation of a viable mutant allele of *IMP3*

**Figure 1:**
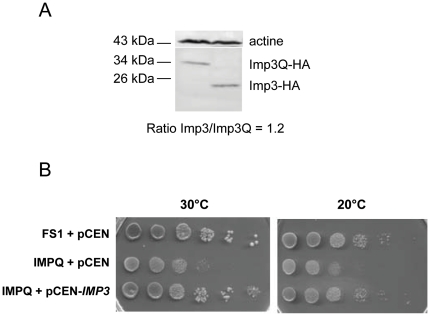
Imp3Qp expression and phenotypes of the IMPQ strain. (A) Western blot analysis of the Imp3Q and Imp3 protein levels. HA-tagged proteins were expressed in the wild-type FS1 strain. (B) IMPQ growth phenotypes: serial dilutions of FS1 and IMPQ cultures grown on YPD at 20°C and 30°C for 3 days.

In the course of studying translation termination accuracy at the stop codon of the essential *IMP3* gene of *S. cerevisiae*, we constructed a mutant allele of *IMP3* where the endogenous TAA stop codon was changed to a glutamine codon (CAA). The resulting mutant allele (*imp3Q*) is thus lengthened 240 nucleotides downstream until the following in frame stop codon. To construct the corresponding strain (IMPQ strain), the mutant allele was first obtained on a vector by site-directed mutagenesis of wild-type *IMP3* gene and was then inserted at the endogenous locus of a FS1 strain. The resulting IMPQ strain is viable and produced a C-terminal elongated Imp3 protein with an 80 amino acid extension (Imp3Qp). No functional site or known protein domain was identified in the extension. We verified by western blotting of HA-tagged proteins that *imp3Q* mutation had no impact on the stability of the protein, since an equivalent amount of wild-type and mutant Imp3 proteins was observed (Figure 1A). Expression of the mutant allele is compatible with cell growth (3h20 doubling time at 30°C in rich and minimum media versus, respectively, 2h and 2h20 for FS1), and leads to a cold-sensitivity at 20°C (Figure 1B). Growth phenotypes of the IMPQ strain are corrected by expression of a wild-type *IMP3* allele (complemented IMPQ strain; 2h30 doubling time at 30°C in minimum medium and Figure 1B). To our knowledge this is the first description of a viable mutant allele of *IMP3*.

### The IMPQ strain is defective in small ribosomal subunit formation

**Figure 2:**
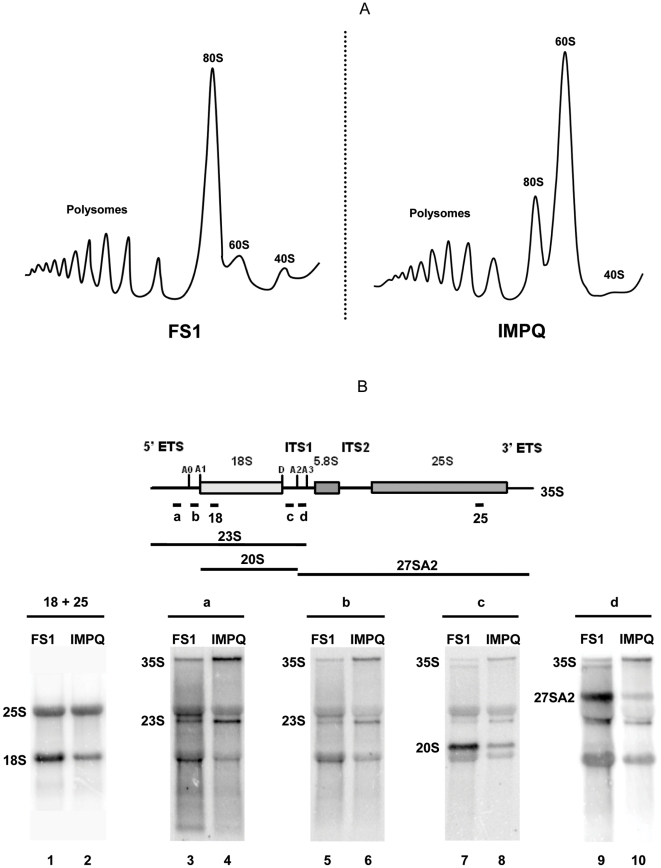
Polysome profile and pre-rRNA processing defects of the IMPQ strain. (**A**) Polysome profile of the FS1 and IMPQ strains. Equivalent amounts of polysome extracts were analyzed by sucrose gradients in each case. The positions of 40S and 60S subunits are indicated as well as the monosomes (80S) and polysome peaks (**B**) Northern blot analyses of pre-rRNA and mature rRNA in the FS1 (lanes 1, 3, 5, 7, 9) and IMPQ (lanes 2, 4, 6, 8, 10) strains. The 35S rRNA precursor is schematically represented on the top of the figure, with the position of all oligonucleotides used as probes: (18)+(25) lanes 1, 2; (a) lanes 3, 4; (b) lanes 5, 6; (c) lanes 7, 8; (d) lanes 9, 10. The 23S, 20S and 27SA_2_ species are represented by bars. Non-specific signals were obtained for 18S and 25S RNA in (a), (b), (c) and (d) blots.

Previous works involving the Imp3 protein showed that a depletion of this essential component of the SSU processome led to important defects in pre-rRNA processing [4]. To determine whether it was the case of the IMPQ strain, we examined ribosome biogenesis by first looking at polysome profiles. The cell ribosome population was analysed on sucrose gradients and compared to those produced by the FS1 strain. Results clearly showed that in the IMPQ strain, free 40S subunits were less detectable while there was a large excess of free 60S subunits (Figure 2A). The 80S monosome population was also decreased in IMPQ strain, but surprisingly, overall polysome level was rather similar between the two strains.

Next, the total amount of 60S and 40S subunits was analysed in the wild-type FS1, the mutant IMPQ and the complemented IMPQ strains by dissociating the 80S and polysome complexes into free subunits. The ratio 60S/40S was 2.8 in the wild-type, whereas in IMPQ strain, the ratio was 5.6 (Figure S1). These results confirmed the *imp3Q* mutation led to a reduction in the steady-state level of 40S subunits. Expression of the wild-type *IMP3* allele in the IMPQ strain (complemented IMPQ strain) corrected the defect in 40S subunit production, restoring a wild-type ribosomal subunit ratio (60S/40S ratio of 2.7, Figure S1).

The IMPQ strain may display a delay in small subunit biogenesis, resulting in less free 40S and an increase in free 60S. Despite this defect enough translating ribosomes are generated to sustain cell growth. This indicates that, 40S subunits are efficiently associated to 60S subunits to produce translating ribosomes. Considering the slower generation time of the IMPQ strain, the observation might suggest that other cellular defects (see below) participate in growth impairment of the strain.

### The IMPQ strain shows a cleavage delay at the A_0_, A_1_ and A_2_ sites

To determine further the ribosomal defect of the IMPQ strain, we monitored Northern blot analyses to visualise rRNA precursor species. Experiments were done on total RNA extracted from FS1 and IMPQ strains and probed with specific oligonucleotides to discriminate pre-rRNA precursors (Figure 2B). Consistent with the observed 40S synthesis defect, the IMPQ strain showed a noteworthy decrease (2.5 fold) in 18S rRNA steady state level compared to the FS1 strain, whereas levels of 25S rRNA were equivalent in both strains (Figure 2B, lanes 1 and 2). Probing with oligonucleotides (a) or (b) (complementary to sequences upstream the A_0_ site or between sites A_0_ and A_1_, respectively; Figure 2B, lanes 3–6) evidenced an accumulation of the 35S precursor and of the aberrant 23S product, which results from a cleavage defect at sites A_0_, A_1_ and A_2_. Probing with oligonucleotide (c) specific to the ITS1 region and located upstream the A_2_ site, revealed that the level of the 20S pre-rRNA was strongly reduced in the IMPQ strain (Figure 2B, lanes 7 and 8), which correlated to the decrease in mature 18S rRNA. Finally, probing with oligonucleotide (d), specific to the ITS1 region but located between the A_2_ and A_3_ sites, showed that the 27SA_2_ pre-rRNA was as well decreased in the IMPQ strain (Figure 2B, lanes 9 and 10). This was expected since 20S and 27SA_2_ pre-rRNA are the two by-products of the A_2_ cleavage. In summary, the data demonstrated that the Imp3Q protein affected the pre-rRNA maturation process by specifically reducing cleavages at the A_0_, A_1_ and A_2_ sites.

Altogether, results indicated that the expression of the modified Imp3Q protein leads to ribosome biogenesis defects similar to those obtained after depletion of the Imp3 protein [4]. Nonetheless the impairments were not lethal for the IMPQ strain, allowing to investigate other strain phenotypes.

### The U3 snoRNA is stable in the IMPQ strain

The endonucleolytic cleavages at the A_0_, A_1_, and A_2_ sites require formation of two short duplexes between the U3 snoRNA and the pre-RNA [12], [13]. Although neither Imp3p, nor Imp4p was required for maintenance of U3 snoRNA integrity [4], we verified that its levels remained unchanged in the IMPQ strain. For this, total RNA isolated from FS1 (Δ*imp3*::*ADE2 *+ pCEN-*IMP3*) and FS1 (Δ*imp3*::*ADE2 *+ pCEN-*imp3Q*) strains were analyzed by Northern blotting with specific U3 snoRNA and the normalizing small cytoplasmic *scR1* RNA probes. The U3 snoRNA/*scR1* ratio was similar in both strains (Figure S2A), thus indicating that *imp3Q* expression did not affect the steady-state level of U3 snoRNA.

### The Imp3Q protein interacts less efficiently with Mpp10p

**Figure 3:**
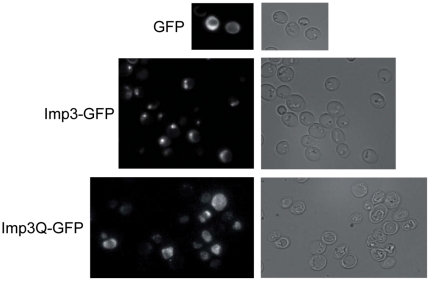
Fluorescence microscopy of the Imp3-GFP and Imp3Q-GFP fusions. Localization of both fusions was observed in the wild-type strain grown on plasmid-selective minimum medium at 30°C.

The Imp3 protein is known to interact with the essential Mpp10 protein to form the Imp3p-Mpp10p-Imp4p core complex of the SSU processome [4], [7]. We thus analysed the interaction of the Imp3Q mutant protein with the Mpp10p partner using a two-hybrid approach. The entire *IMP3* and *IMPQ* reading frames were fused to the binding domain of Gal4p (Gal4-BD-IMP3 and Gal4-BD-IMPQ). Two constructs were made for the fusion of Mpp10p with the Gal4p activating domain, as Lee and Baserga [4] described that amino acids 242 to 498 of Mpp10p are sufficient for interaction with Imp3p.We reproduced the previously observed interactions between Imp3p and Mpp10-5p (aa 1–498) or Mpp10-6p (aa 242–498), but no Mpp10p interaction was revealed with the Imp3Q protein (Figure S2B). Still, the IMPQ strain being viable, interaction between these two essential partners involved in ribosome biogenesis might be below detection limits. In human, the nucleolar accumulation of Imp3p, Imp4p and Mpp10p was dependent on formation of the ternary complex [7]. We thus constructed and observed the location of Imp3-GFP and Imp3Q-GFP fusions in the wild-type strain. The Imp3Q protein appeared less efficiently localized to the nucleus than the Imp3 protein (Figure 3). Moreover, the modified Mpp10p-Imp3Qp interaction might be responsible for the ribosome biogenesis defects described for the IMPQ strain.

### The IMPQ strain displays an elevated rate of +1 frameshifting

**Figure 4:**
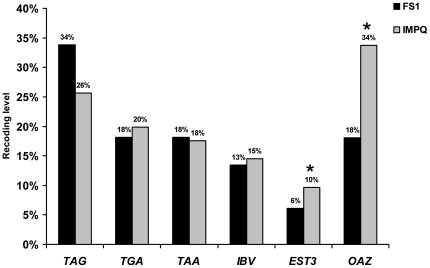
Translational accuracy in the IMPQ strain. The median recoding efficiency of at least five independent experiments is indicated for each target sequence assayed (*TAG*, *TGA* and *TAA* for readthrough, *IBV* (Avian infectious bronchitis virus) for −1 frameshifting, *EST3* and *OAZ* for +1 frameshifting). Results significantly different between the IMPQ and FS1 strains are marked by an asterisk (*p*-value<0.0001).

We then investigated whether such ribosome biogenesis defects has any impact on translation accuracy. The efficiency of translation termination at the three stop codons and of +1 or −1 frameshifting was analysed in the FS1 and IMPQ strains using a set of reporter plasmids carrying the corresponding target sequences. These plasmids contained the readthrough motif from the Tobacco Mosaic Virus, or the −1 frameshift IBV (Infectious Bronchitis Virus) sequence, or the +1 frameshift *EST3* (“Ever Shorter Telomere 3”) and *OAZ1* (Ornithine decarboxylase AntiZyme) sequences [18]. Each strain was transformed with the reporter plasmids and enzyme activities were measured. The given values correspond to the median of at least five measurements (Figure 4). Statistical analyses using the Mann-Whitney non-parametric test were performed. No significant effect on translational readthrough was observed in the IMPQ strain whatever the stop codon (*p-*value *TAG* = 0.797, *TGA* = 0.898 and *TAA* = 0.898), neither on −1 frameshifting (*p-*value *IBV* = 0.878). However the +1 frameshifting rate increased in the IMPQ strain. Recoding efficiency at both the *EST3* and *OAZ1* sequences are about two fold higher than in the FS1 strain (*p-*value<0.0001). Although the +1 frameshifting seemed clearly affected in the IMPQ strain, the underlying mechanisms are not known.

### The IMPQ strain is sensitive to DNA damaging agents

Strains with a modified translation accuracy often displayed aminoglycoside sensitivity. We thus investigated the resistance/sensitivity of the IMPQ strain against various antibiotics. We tested antibiotics targeting different translation steps, such as the aminoglycosides: G418, paromomycin, ribostamycin, tobramycin, neomycin and kanamycin [19], as well as DNA damaging agents like bleomycin and phleomycin [20].

**Figure 5:**
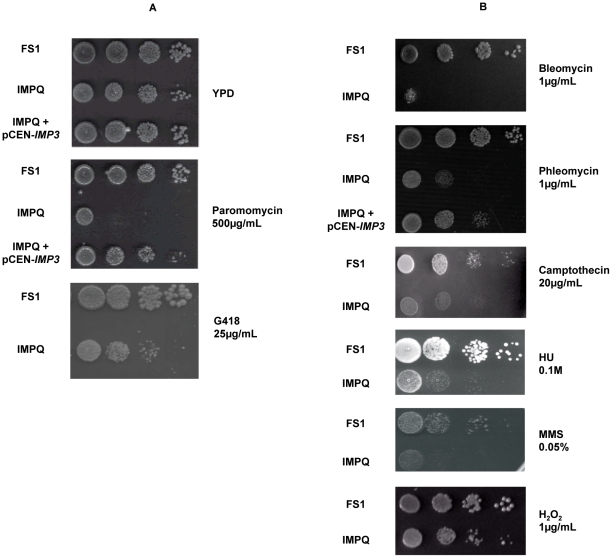
Drug sensitivity of the IMPQ strain. Growth of the FS1 and IMPQ strains is shown on YPD plates containing various concentrations of (**A**) aminoglycosides (Paromomycin 500 µg/mL, G418 25 µg/mL), or (**B**) DNA damaging agents (Bleomycin 1 µg/mL, Phleomycin 1 µg/mL, Camptothecin 20 µg/mL, Hydroxyurea (HU) 0.1M, Methyl methanesulfonate (MMS) 0.5%, H_2_O_2_ 1 µg/mL). Culture of the IMPQ strain complemented by pCEN-*IMP3* was also assayed on phleomycin (1 µg/mL).

Exponentially growing FS1 and IMPQ strains were serially diluted and spotted onto rich medium containing different concentrations of each indicated antibiotics (Figure 5). Results highlighted two interesting points. First, the IMPQ strain appeared to be sensitive to the G418 and paromomycin aminoglycosides (Figure 5A). At 250 µg/mL paromomycin, growth of the IMPQ strain is already inhibited (data not shown), while at 500 µg/mL, no growth was observed. In the same conditions, growth of the FS1 strain was not impaired. Second, an unexpected high sensitivity to bleomycin and to the closely related phleomycin was observed for the IMPQ strain (Figure 5B). The former result was not surprising as G418 and paromomycin target the ribosome and the IMPQ strain harboured defects in the ribosome biogenesis. The latter was more intriguing, since bleomycin and phleomycin are anti-tumoral agents known to induce various types of DNA damages. To verify the antibiotic sensitivity phenotypes were directly related to the expression of the mutant *imp3Q* allele, the IMPQ strain was transformed with a plasmid-encoded wild-type *IMP3* gene, and the phleomycin assay was reproduced. As shown on Figure 5B, the presence of a wild-type Imp3 protein restored antibiotic resistance of the IMPQ mutant strain. It should be noted that the assays were made on rich medium, so a non negligible proportion of IMPQ cells may have lost the pCEN-*IMP3* plasmid, explaining why a wild-type growth was not totally restored. Indeed each time the complementation was assayed under selection to retain the plasmid (arginine 0.2% minimum medium without uracil), the IMPQ sensitivity phenotype was quite fully corrected (Figure S3). We can therefore conclude the antibiotic sensitivity phenotypes of the IMPQ strain were directly dependent on the partial loss of function of the Imp3 protein.

### The IMPQ strain is impaired in DNA repair

The bleomycin is known to induce a narrow set of oxidative DNA lesions : i) formation of apuric/apyrimidic sites (AP sites), ii) single strand breaks and iii) bi-stranded DNA lesions at certain specific sequences which are then converted into iv) double-stranded breaks [20]. The antibiotic sensitivity of the IMPQ strain suggested that Imp3p might be involved, directly or indirectly, in repair of the DNA lesions induced by the bleomycin treatment. As each type of lesions is corrected by distinct mechanisms, we tried to determine more precisely which of them was affected in the IMPQ strain. We therefore assayed other chemical agents inducing more specific lesions on the DNA. First, we used the camptothecin drug, which stabilizes the topoisomerase I-DNA cleavage intermediates and thereby gives rise to double-stranded breaks quite similar to those obtained after bleomycin treatment [21]. Several camptothecin concentrations were assayed, and sensitivity of the IMPQ strain was revealed at high concentration (20 µg/mL, Figure 5B). In a same way, the IMPQ strain was slightly sensitive to the alkylating agent methyl methanesulfonate (MMS) [22] and the ribonucleotide reductase inhibitor hydroxyurea (HU), which cause replication fork stalling [23] (Figure 5B). We then tested the sensitivity to various concentrations of hydrogen peroxide (H_2_O_2_), which produces highly reactive hydroxyl radical inducing many DNA modifications, such as release of free bases, single strand breaks, sugar modifications, or AP sites [24]. In this case, we observed no growth differences between the IMPQ and FS1 strains (Figure 5B). Altogether, these results suggest that Imp3p might be involved in reparation of DNA double-stranded breaks.

### The IMPQ strain displays lengthened telomeres

**Figure 6:**
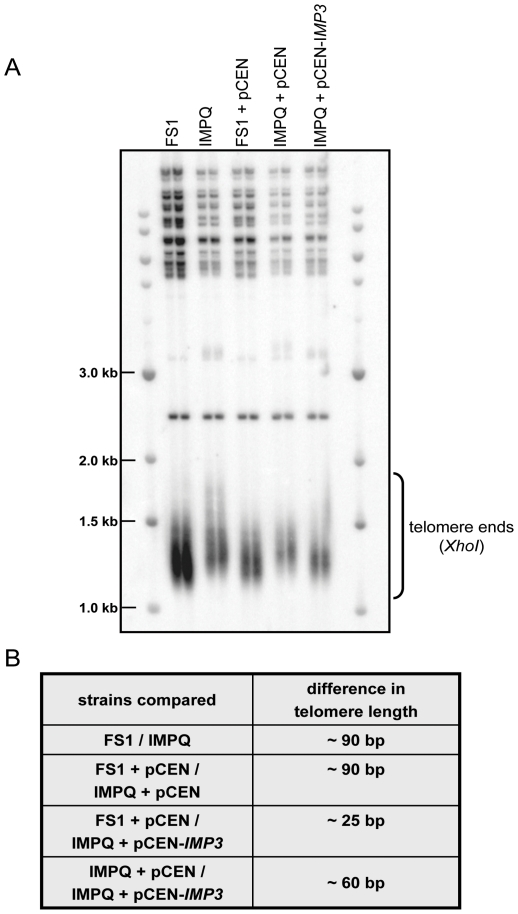
Analysis of telomere length in wild-type and IMPQ strains. (**A**) Genomic DNA from two independent clones digested with *XhoI* and probed with telomere-specific probe, detecting TG_1–3_ repeats. Telomere ends are bracketed, the 1 kb DNA ladder (NEB) was migrated in the first and last lines, and internal migration control is the sharp band of 2.5 kb. FS1 and IMPQ strains were grown in YPD medium overnight at 30°C, FS1 and IMPQ strains harbouring pCEN and pCEN-*IMP3* plasmids were grown in CSM-URA medium in the same conditions. (**B**) Difference in telomere length is indicated between various strains.

Recent works concerning the Imp4 protein suggested its potential role in telomere metabolism [16], [17]. To test whether Imp3p was also implicated, we analysed telomere lengths in the FS1 and IMPQ strains. Genomic DNA was extracted from both strains, *Xho*I-digested, and submitted to a Southern blot analysis with specific TG_1–3_ telomeric probes. *Xho*I sites are present within the subtelomeric regions of most of the Y′-containing telomeres [25]. After complete digestion, probing the telomere repeats shows a ∼1.2 kb DNA corresponding to the telomere extremities. Variation in the numbers of telomeric repeats is easily assessed on agarose gel as inactivation of factors mediating telomere elongation shows shorter telomere fragments. Indeed, as expected the deletion of the subunit of yeast telomerase Est3p [26] resulted in telomere shortening about ∼200 bp in the FS1 strain (Figure S4). By contrast, the IMPQ strain presented significantly longer telomeres (about 90–100 bp) than the FS1 strain (Figure 6 and S4), suggesting that Imp3 protein could inhibit telomere lengthening. Expression of the wild-type *IMP3* allele restored normal telomere length in the IMPQ strain.

As previously mentioned, the IMPQ strain displayed increased expression of a reporter gene containing the *EST3* +1 frameshift. Thus, it is tempting to speculate that the role of IMPQ on telomere length would be related to Est3p overproduction. To test this idea, *EST3* gene was deleted both in the FS1 and IMPQ strains. Strikingly, though telomere sizes were reduced in *est3*Δ strains, similar relative increases of telomere lengths were observed in IMPQ *est3*Δ and IMPQ strains when compared to *est3*Δ and FS1, respectively (about 80 bp, Figure 6). Overall, these data suggest that Imp3p controls telomere elongation independently of Est3p expression.

## Discussion

Here, we took advantage of a viable mutant allele of the essential *IMP3* gene that permits cell growth to investigate the Imp3p functions. The mutant IMPQ strain harboured relevant growth and functional defects. The IMPQ strain appeared to be cold sensitive and its growth was lightly slowed compared to the wild-type strain. Both phenotypes were already reported for mutations affecting ribosome assembly, or pre-rRNA processing [11], notably with a Mpp10p mutant [7]. As expected, the IMPQ strain was impaired for ribosome biogenesis, presenting a decrease of free 40S ribosomal subunits and less mature 18S rRNA. Results also suggested Imp3Qp interacted less efficiently with Mpp10p, reducing the protein import into the nucleus, which could indeed produce the observed ribosome biogenesis defects.

We then investigated the impact of the mutation on protein translation accuracy. No effect was detected on stop codon readthrough and −1 frameshifting, but +1 frameshifting was significantly increased. The effect was observed with two programmed +1 frameshift sequences using either a rare sense codon (*EST3*) or a shifty stop codon (*OAZ1*) as a stimulatory element [27], and are thus controlled by different mechanisms. This indicates that the effect likely concerned a common mechanism. Since for both sequences, stimulatory elements in the A site of the ribosome induce pausing, one may suggest the ribosome pausing time is increased in presence of Imp3Qp, due to some expected ribosomal alterations occurring in the strain, as previously inferred for −1 frameshift signals and delayed rRNA processing [28]. Actually, the pre-rRNA cleavage defects might produce aberrant 40S small subunits altered in frame maintenance. Yet, we cannot exclude an unknown function of the Imp3 protein being impaired in the IMPQ strain. The IMPQ strain also appeared to be highly sensitive to G418 and paromomycin, two aminoglycosides targeting the decoding center of the 18S rRNA in the small subunit. However, the IMPQ strain was not sensitive to the other tested aminoglycosides, the effect thus seemed to be specific to a subclass of aminoglycosides.

The IMPQ strain was also highly sensitive to bleomycin and to the structurally related phleomycin, which produce different kinds of toxic DNA lesions [20]. Sensitivity to an elevated concentration of camptothecin, HU and MMS, but not to H_2_O_2_ was revealed. All phenotypes suggest an involvement of Imp3p in double strand break (DSB) repair. To date the Imp3 protein has never been observed participating in an extra-ribosomal biogenesis pathway. However, recently, Imp4p partner of Imp3p in the Mpp10 subcomplex of the SSU processome was related to DNA DSB repair [29] and telomere length control [16]; [17]. This and the present work highlighted the putative role of the whole Mpp10 subcomplex in DNA repair processes.

Furthermore, a recent genome-scale study of synthetic genetic interactions in yeast revealed a connection between Mpp10p and several proteins implicated in telomere maintenance and silencing (Stm1p, Upf3p, Nmd2p, Dpb4p) [30]. Meaningfully, each partner of the Imp3-Mpp10-Imp4 subcomplex was independently, and by different approaches, linked to the telomere metabolism. Nevertheless, we can not exclude indirect effects due to reduced translation and further investigations are needed to elucidate this issue.

An indirect role of Imp3p in telomere length control was anticipated through the increase of the *EST3* +1 frameshifting, which produces more functional subunits of the telomerase. Although western blot analysis confirmed that the Est3 protein was slightly (∼10%) more abundant in the IMPQ strain compared to the FS1 strain (data not shown), it could not be responsible for the telomere length difference. Yet, telomeres were still longer in the IMPQ strain deleted for the *EST3* gene. We also tested a strain deleted for Upf1p (“Up Frameshift 1 protein”), a helicase implicated in non-sense mediated mRNA decay, which displays an increased shifty stop-dependent +1 frameshifting [31]. The strain was observed to be resistant to phleomycin (data not shown) and is described for having telomere shortening [32]. Results thus strongly argued for a role of Imp3p in telomere metabolism and antibiotic sensitivity independently of the telomerase or increased +1 frameshifting activities.

Yeast telomeres are transcribed into telomeric repeat-containing RNA (TERRA) [33], which inhibit telomerase action *via* a DNA/RNA hybrid [34]. We could infer a role of Imp3p, or a larger snoRNP particle, in processing of these TERRA molecules, or of the *TLC1* telomerase RNA.

The role of Imp3p in pre-rRNA processing is evolutionary conserved between yeast and mammals. It might therefore be interesting to inquire about functions of Imp3 proteins in DNA repair and telomere metabolism in higher organisms. It might be a general property of RNA-processing proteins to have dual functions in the nucleus.

## Materials and Methods

### Strains and genetic methods

The *S. cerevisiae* strains were constructed in the FS1 background (MAT α *ade2-592* (frameshift) *lys2Δ201 leu2-3,112 his3Δ-200 ura3-52*), which is derived from the Y349 strain [35]. For the two-hybrid analysis, the pJ69-4a and pJ69-4α strains were used (MAT *a* or *α trp1-901 leu2-3*, *112 ura3-52 his3Δ-200 gal4Δ gal80Δ GAL2-ADE2 LYS2::GAL1-HIS3 met2::GAL7-lacZ*) [36]. Strains were cultivated in standard rich (YPD) or synthetic (SC) media for yeast [37]. Appropriate amounts of antibiotics, amino acids and bases were added when necessary. DNA transformation of lithium acetate-treated yeast cells was performed as described in [38]. For plasmid shuffling, a selective medium containing 0.25% 5-fluoroorotic acid (5-FOA, Fermentas) was used. For microscopic observation, yeast strains were grown in selective minimum medium to an OD_600_ = 1–2, examined under a fluorescence microscope (Zeiss Axioplan), and photographed by a CCD-RTEA-CCD1317 camera (Princeton Instruments). For western blotting, total proteins were extracted from cells cultivated in appropriate selective medium, submitted to SDS-PAGE electrophoresis, and HA-tagged Imp3 proteins were revealed with the anti-HA 3F10 antibody from Roche, as described in [39].

### Plasmids and oligonucleotides

All the oligonucleotides used are presented in Table S1.

The *IMP3* gene was first cloned under its own promoter (fragment amplified between IMP_785_ and IMP_1924_ oligonucleotides) into the pUC19 and the centromeric *URA3* pFL38 vectors (pCEN-*IMP3*). The pUC19-*IMPQ* was obtained by QuickChange site-directed mutagenesis (Stratagene) of the *IMP3* stop codon using the IMPQw and IMPQc oligonucleotides. Vectors pYEF1-*IMP3* and pYEF1-*IMPQ* expressed N-terminal HA-tagged Imp3 proteins in presence of galactose (PCR fragments amplified with Imp3NotNter and Imp3EcoCter oligonucleotides). For the two-hybrid analysis, the *IMP3* and *IMPQ* reading frames were PCR-amplified, respectively, from pUC19-*IMP3* with the IMP3-N-ClaI/IMP3-C-PstI oligonucleotides, or from pUC19-*IMPQ* with the IMP3-N-ClaI/IMPQ-C-PstI oligonucleotides, and cloned in frame into the correspondingly digested pGAD-C3 (2 µ, *LEU2*) or pGBDU-C3 (2 µ, *URA3*) vectors [36]. The *MPP10-5* and *MPP10-6* fragments of the *MPP10* reading frame were generated by PCR from the FS1 genomic DNA using either the MPP10-5-N-BamHI/MPP10-6-C-SalI, or the MPP10-6-N-BamHI/MPP10-6-C-SalI oligonucleotide couples, respectively, and cloned in frame into the pGAD-C3 vector. The IMP3-N-BamH1 and IMP3-C-EcoRI or IMPQ-C-EcoR1 oligonucleotides amplified, respectively, the *IMP3* or *IMP3Q* alleles for cloning into the corresponding sites of the yEGFP3-C-FUS vector (CEN, *HIS3*), generating GFP C-terminal fusions.

### Strain construction

The IMPQ strain was generated by a two-step strategy described in Data S1, where the FS1Δ*imp3:*:*ADE2*+pCEN-*IMP3* strain was transformed with the *imp3Q* allele, looking for a viable clone after pCEN-*IMP3* shuffling.

Deletion of the *EST3* gene by the KanMX6 cassette in the FS1 and IMPQ strains (FS1 *est3*Δ and IMPQ *est3*Δ) was done by one-step PCR-mediated technique [40]. Positive clones were selected on 200mg/L G418 and proper integration was verified by PCR.

### Polysome and ribosomal subunit analyses

Polysomes from the IMPQ and FS1 strains were prepared according to [41], and briefly described in Data S1. Cultures were grown at 30°C in glucose rich medium and harvested at an OD_600_ of 0.8–0.9. Gradient analyses were performed with an absorbance detector (Isco, Inc) and continuously monitored at A_254nm_.

To determine the 60S/40S ratio, monosomes and polysomes complexes were completely dissociated into subunits by performing the extractions in low-Mg^2+^ buffer (50 mM Tris-HCl, pH 7.4; 50 mM NaCl; and 1 mM DTT) and loaded on to a 10%–30% sucrose gradient (see also Data S1).

### Northern blot analyses

FS1 and IMPQ strains were grown at 30°C in selective minimum medium to an OD_600_ = 1–2, and total RNA extracted as described in [42]. High-molecular-weight RNA (10 µg total RNA) were submitted to electrophoresis on a 0.9% agarose-formaldehyde gel [43], and overnight transferred to positively charged Hybond™-N^+^ membranes (GE Healthcare) by capillary elution with 20× SSC. Oligonucleotides were 5′-labeled with [γ^32^P]-ATP, purified on microspin G50 columns, and used for Northern hybridizations [44]. Signals were visualized on a *STORM imager* (Molecular Dynamics) and quantified with the *ImageQuant* software.

For the U3 snoRNA Northern blot analysis, RNA samples (4 µg total RNA) were fractionated on a 6% polyacrylamide gel containing 7 M urea, and electro-transferred onto membrane in 1× TAE. Hybridizations were done as described in [44], with the (U3 snoRNA) and (SCR1) oligonucleotides. For quantification, signal intensity was measured with the TINA program (Fuji Photo Film).

### Two-hybrid analysis

The pGAD (empty pGAD, pGAD-*IMP3*, pGAD-*IMPQ*, pGAD-*MPP10-5* and pGAD-*MPP10-6*) and pGBDU based plasmids (empty pGBDU, pGBDU-*IMP3* and pGBDU-*IMPQ*) were transformed into the pJ69-4α and pJ69-4a strains, respectively. Transformants mating was done in different pairwise combinations, overnight at 30°C on YPD plates. Diploids were selected on uracil and leucine omission medium. Interactions were visualized by expression of the *HIS3* or *ADE2* reporter genes on minus adenine/uracile/leucine or 10mM 3-amino-1,2,4-triazole minus histidine/uracile/leucine plates, respectively. As negative control, diploids bearing empty vectors were analysed, and no reporter gene expression was observed.

### Quantification of recoding efficiency

The FS1 and IMPQ strains were transformed with a set of dual reporter plasmids, carrying a *lacZ* – recoding sequence – *luc* gene fusion under a constitutive promoter [45]. The recoding sequences corresponded to −1/+1 frameshifting or stop codon readthrough sequences, allowing to measure the translation accuracy [18]. Recoding efficiency at 30°C was expressed as the percent of luciferase/β-galactosidase activity ratio compared to an in-frame fusion construct [45]. Such constructs eliminated any differences in mRNA abundance or translation initiation efficiency. Data were analysed with the Mann-Whitney non-parametric statistical test (XLSTAT 2007 software).

### Temperature and drug sensitivity assays

FS1 and IMPQ strains were cultivated in rich medium and serial dilutions were spotted on either YPD plates at 20°C and 30°C, or drug containing YPD plates at 30°C, in order to assess the temperature or drug sensitivities, respectively. The assayed drugs and concentrations are indicated in Data S1.

### Telomere length analysis

Total DNA was isolated from cells grown overnight in YPD (A_600_ = 2.0). 50–100 µg DNA was digested with *Xho*I (NEB) overnight at 37°C and run on 25-cm-long 0.8% agarose gels for 18 h at 100V. Restriction-digested fragments, containing *S. cerevisiae* telomeric repeats were loaded with digested DNA as a size control. Southern blotting was performed using Amersham Hybond™-XL membranes (GE Healthcare), and probed with radiolabeled (dCTP, α^32^P, Exo (-) Klenow Polymerase from Stratagene) telomeric probe containing TG_1–3_ repeats. Hybridizations were done in PerfectHyb™ Plus Hybridization Buffer (Sigma) at 65°C. The average telomeric length was estimated for each lane by calculating the distance from the peak of signal intensity of the telomere band against the position of the added internal size standard (Quantity One 4.6.5 Basic, Biorad).

## Supporting Information

Figure S1 **Relative abundance of the 40S and 60S subunits.** Determination of the ribosomal subunit ratio in the wild-type FS1 strain, the mutant IMPQ strain, and the complemented IMPQ strain carrying the pCEN-*IMP3* vector.
(TIF)

Figure S2 **Levels of the U3 snoRNA in the FS1 and IMPQ strains, and interaction between the Imp3 and Mpp10 proteins.** (**A**) Northern blot analysis of the U3 snoRNA. Positions of the U3 and SCR1 RNA are indicated, as well as the U3/SCR1 ratio in percent. (**B**) Two-hybrid analysis. The growth of diploid strains carrying the indicated vectors is presented on minimal medium lacking leucine, uracile and adenine.
(TIF)

Figure S3 **Drug sensitivity of the IMPQ strain.** Growth of the FS1 and IMPQ strains transformed by an empty pCEN-*URA3* vector (pFL38 vector), as well as the complemented IMPQ strain carrying the pCEN-*IMP3* vector, is shown on arginine 0.2% minimum medium plates without uracil, and containing various concentrations of DNA damaging agents (Phleomycin 1 µg/mL, Bleomycin 1 µg/mL, Camptothecin 20 µg/mL, Hydroxyurea (HU) 0.1M, H_2_O_2_ 1 µg/mL).
(TIF)

Figure S4 **Analysis of telomere length.** (**A**) The 1 kb DNA ladder is shown on the right panel. The upper signal on the left panel is an internal size reference. Lower bands are XhoI-digested telomeric DNA fragments revealed with specific telomeric probe. At least two independent DNA extractions are shown for each strain. *est3*Δ corresponds to strains deleted for *EST3*. (**B**) Difference in telomere length is indicated between various strains.
(TIF)

Table S1 **List of the oligonucleotides used in the study.** Restriction sites are underlined. The CAA mutation of the *IMP3* stop codon is in bold.
(DOC)

Data S1 (DOC)
