# Correction: A Viable Hypomorphic Allele of the Essential *IMP3* Gene Reveals Novel Protein Functions in *Saccharomyces cerevisiae*

**DOI:** 10.1371/annotation/18c67950-0fcc-4ba8-aa59-5d6a3e060c68

**Published:** 2011-05-31

**Authors:** Bruno Cosnier, Marta Kwapisz, Isabelle Hatin, Olivier Namy, Sylvie Hermann-Le Denmat, Antonin Morillon, Jean-Pierre Rousset, Céline Fabret

The 5th author's name is incorrect in the citation. The correct citation is: 

Cosnier B, Kwapisz M, Hatin I, Namy O, Hermann-Le Denmat S, et al. (2011) A Viable Hypomorphic Allele of the Essential IMP3 Gene Reveals Novel Protein Functions in Saccharomyces cerevisiae. PLoS ONE 6(4): e19500. doi:10.1371/journal.pone.0019500 

