# Supplementary figures and images for: A Viable Hypomorphic Allele of the Essential IMP3 Gene Reveals Novel Protein Functions in Saccharomyces cerevisiae
Source: PLoS One. 2011 Apr 29;6(4):e19500. doi: 10.1371/journal.pone.0019500 (PMC3084874; doi:10.1371/journal.pone.0019500)

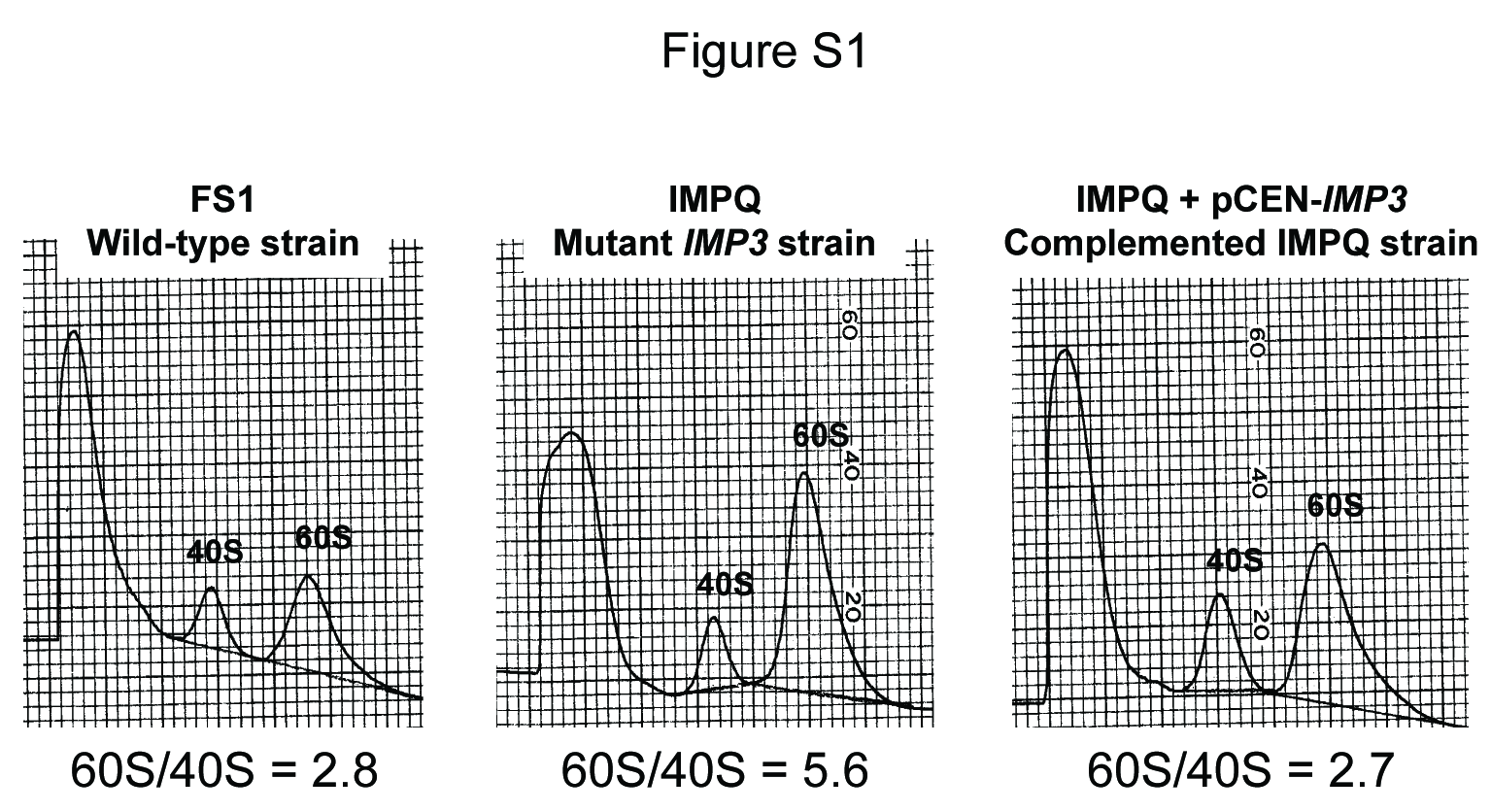

Supplement: Figure S1 — Relative abundance of the 40S and 60S subunits. Determination of the ribosomal subunit ratio in the wild-type FS1 strain, the mutant IMPQ strain, and the complemented IMPQ strain carrying the pCEN-IMP3 vector. (TIF) [file pone.0019500.s001.tif]

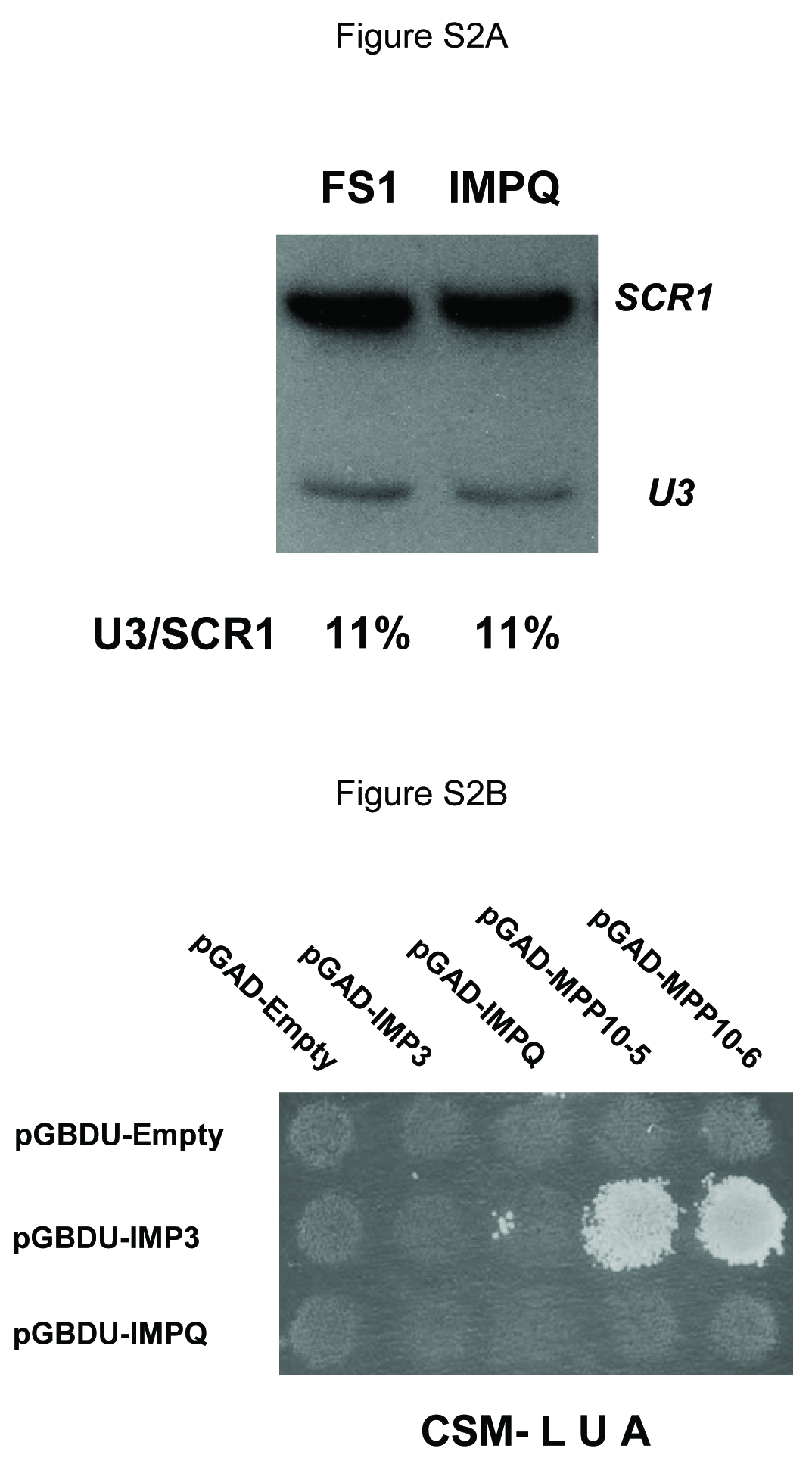

Supplement: Figure S2 — Levels of the U3 snoRNA in the FS1 and IMPQ strains, and interaction between the Imp3 and Mpp10 proteins. (A) Northern blot analysis of the U3 snoRNA. Positions of the U3 and SCR1 RNA are indicated, as well as the U3/SCR1 ratio in percent. (B) Two-hybrid analysis. The growth of diploid strains carrying the indicated vectors is presented on minimal medium lacking leucine, uracile and adenine. (TIF) [file pone.0019500.s002.tif]

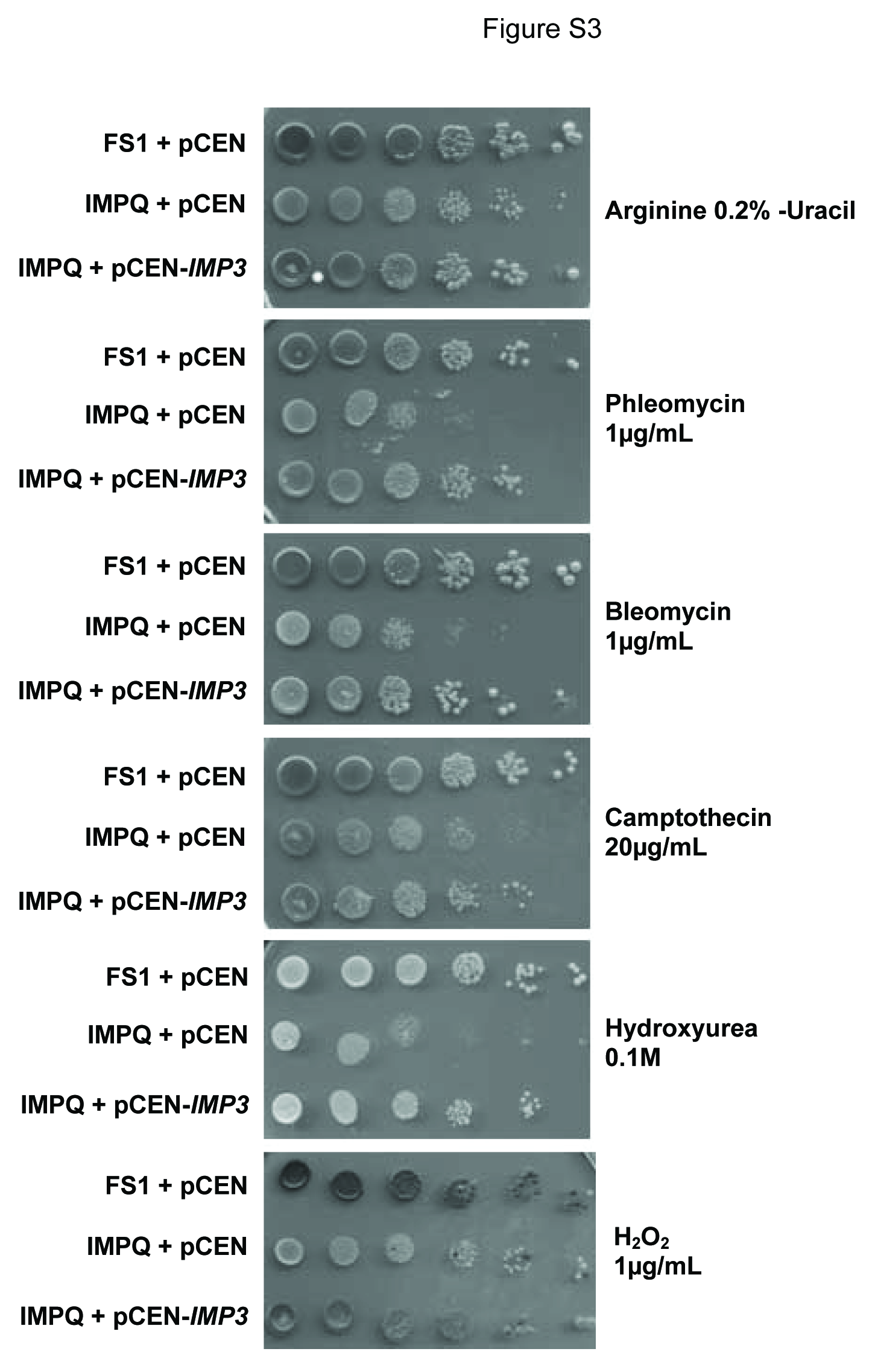

Supplement: Figure S3 — Drug sensitivity of the IMPQ strain. Growth of the FS1 and IMPQ strains transformed by an empty pCEN-URA3 vector (pFL38 vector), as well as the complemented IMPQ strain carrying the pCEN-IMP3 vector, is shown on arginine 0.2% minimum medium plates without uracil, and containing various concentrations of DNA damaging agents (Phleomycin 1 µg/mL, Bleomycin 1 µg/mL, Camptothecin 20 µg/mL, Hydroxyurea (HU) 0.1M, H2O2 1 µg/mL). (TIF) [file pone.0019500.s003.tif]

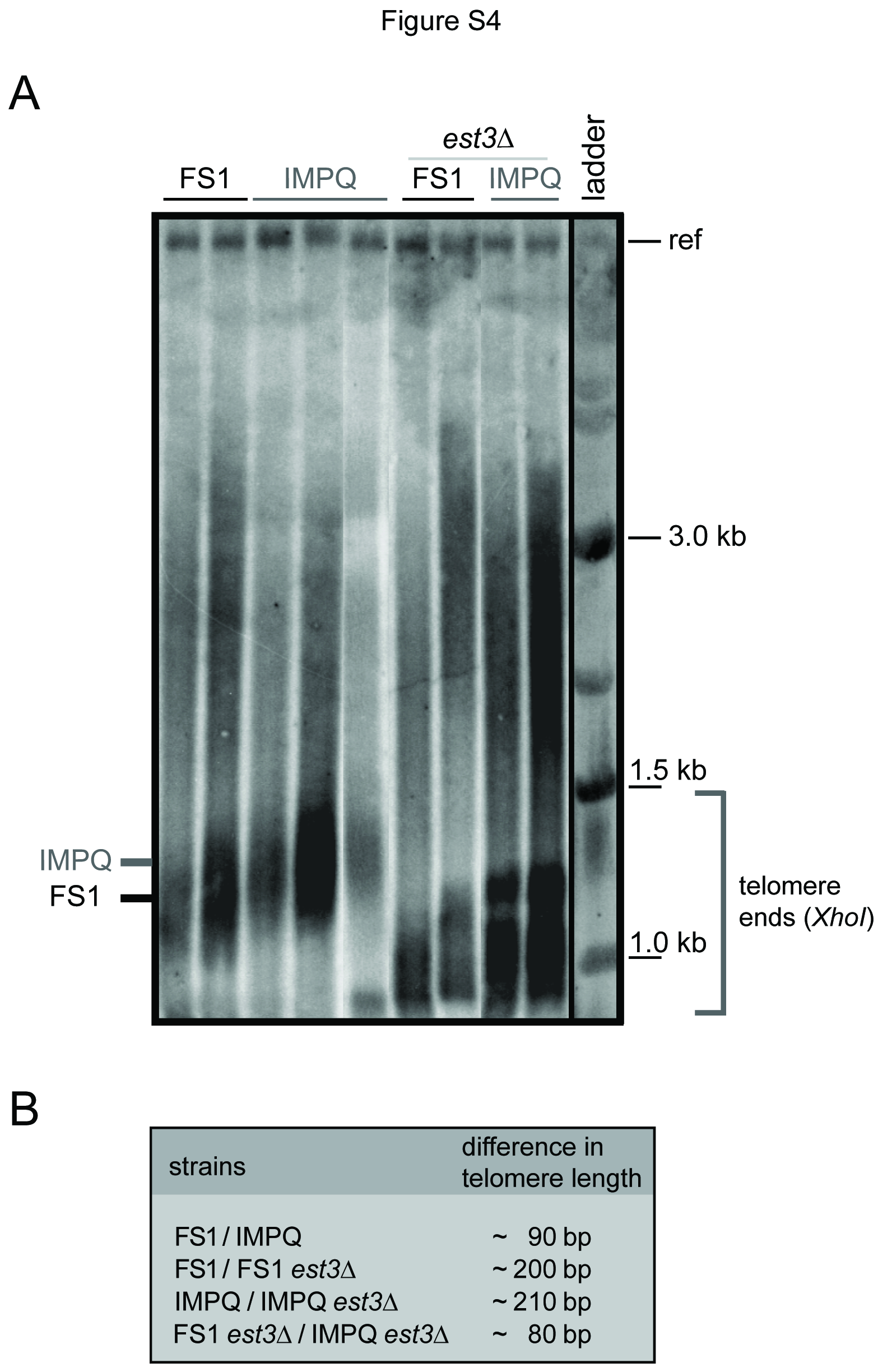

Supplement: Figure S4 — Analysis of telomere length. (A) The 1 kb DNA ladder is shown on the right panel. The upper signal on the left panel is an internal size reference. Lower bands are XhoI-digested telomeric DNA fragments revealed with specific telomeric probe. At least two independent DNA extractions are shown for each strain. est3Δ corresponds to strains deleted for EST3. (B) Difference in telomere length is indicated between various strains. (TIF) [file pone.0019500.s004.tif]
