# Supplementary material for: A Viable Hypomorphic Allele of the Essential IMP3 Gene Reveals Novel Protein Functions in Saccharomyces cerevisiae
Source: PLoS One. 2011 Apr 29;6(4):e19500. doi: 10.1371/journal.pone.0019500 (PMC3084874; doi:10.1371/journal.pone.0019500)
Supplement: Table S1 — List of the oligonucleotides used in the study. Restriction sites are underlined. The CAA mutation of the IMP3 stop codon is in bold. (DOC) [file pone.0019500.s005.doc]

Table S1

| Name | Sequence |
| --- | --- |
| IMP_785_ | 5’-GGATCCTAGCTTTATTGCGTGATCTTCCC-3’ |
| IMP_1924_ | 5’-GGATCCTGAAGTGTAGATAATGAACGGAC-3’ |
| IMP_498_ | 5’-TTATTTTACCGTGCTTTTCATCC-3’ |
| IMP_998_ | 5’-TGGCCATTGGTTGTGATTATGCCTGGTGG-3’ |
| IMP_1765_ | 5’-TCGGCGTCATTTGTTAAATATGC-3’ |
| IMP_2064_ | 5’-TGTATAATTCTCGTGTTAGTGGG-3’ |
| ADE2_216_ | 5’-AACACCAACATAACACTGACATC-3’ |
| ADE2_2319_ | 5’-GGACACCTGTAAGCGTTGATTTC-3’ |
| IMPQw | 5’-GACGATTTTGATTTTTCA**CAA**ATTGACTAC-3’ |
| IMPQc | 5’-GTAGTCAAT**TTG**TGAAAAATCAAAATCGTC-3’ |
| Imp3NotNter | GGGGCGGCCGCCGTTAGAAAACTAAAGC |
| Imp3EcoCter | GGGGAATTCGTTTTAAGGCATATTTAACAAATG |
| (18) | 5’-CATGGCTTAATCTTTGAGAC-3’ |
| (25) | 5’-GCCCGTTCCCTTGGCTGTG-3’ |
| (a) | 5’-TCGGGTCTCTCTGCTGCCGG-3’ |
| (b) | 5’-AACCCACCTATTCCCTC-3’ |
| (c) | 5’-GGTTTTAATTGTCCTATAAC-3’ |
| (d) | 5’-GGGCCCCGATTGCTCGAATG-3’ |
| IMP3-N-BamH1 | 5’-CCGCGGATCCATGGTTAGAAAACTAAAGC-3’ |
| IMP3-C-EcoRI | 5’-CCGGAATTCTGAAAAATCAAAATCGTC-3’ |
| IMPQ-C-EcoRI | 5’-CCGGAATTCAGGCATATTTAACAAATG-3’ |
| IMP3-N-ClaI | 5’-CCATCGATGGTTAGAAAACTAAAGCAT-3’ |
| IMP3-C-PstI | 5’-TCCCCTGCAGTTATGAAAAATCAAAATCGTCG-3’ |
| IMP3Q-C-PstI | 5’-TCCCCTGCAGTTAAGGCATATTTAACAAATGACG-3’ |
| MPP10-5-N-BamHI | 5’-TCCCGGATCCCATGTCAGAACTCTTTGGA-3’ |
| MPP10-6-N-BamHI | 5’-CCGCGGATCCCATGAAAGAACCTGTGAAGAA-3’ |
| MPP10-6-C-SalI | 5’-CCCGGTCGACTCAGACATTGTATATCTCTTGAGG-3’ |
| (U3 snoRNA) | 5’-GTTATGGGACTCATCAACC-3’ |
| (SCR1) | 5’-ATCCCGGCCGCCTCCATCAC-3’ |
