# Supplementary material for: A Viable Hypomorphic Allele of the Essential IMP3 Gene Reveals Novel Protein Functions in Saccharomyces cerevisiae
Source: PLoS One. 2011 Apr 29;6(4):e19500. doi: 10.1371/journal.pone.0019500 (PMC3084874; doi:10.1371/journal.pone.0019500)
Supplement: Data S1 — (DOC) [file pone.0019500.s006.doc]

**Supporting Information**

Two-step strategy for construction of the IMPQ strain

First, a strain with the *IMP3* gene deleted and replaced by the *ADE2* gene was constructed, saved by pCEN-*IMP3*. Second, the *ADE2* gene was deleted and replaced by the mutant *IMP3Q* allele, pCEN-*IMP3* being lost. The non-coding 5’ and 3’ regions of *IMP3* were amplified from the FS1 genomic DNA using, respectively, the IMP_498_ / IMP_998_ (carrying a MscI site) and IMP_1765_ / IMP_2064_ oligonucleotides. After phosphorylation, both fragments were ligated to form the IMP5’3’ fragment, which was then cloned into the SmaI-digested pUC19 vector. The *ADE2* gene was amplified from the Y349 genomic DNA with the ADE_216_ and ADE_2319_ oligonucleotides, and cloned into the MscI site of pUC19-IMP5’3’, resulting in pUC19-Δ*IMP3*::*ADE2*. The “Δ*IMP3*::*ADE2*” cassette was PCR-amplified from the vector using IMP_498_ / IMP_2064_, and then used with pCEN-*IMP3* to transform the FS1 strain. Ura^+^/Ade^+^ white transformants were selected and deletion of the endogenous *IMP3* gene was verified by PCR and sequencing. The resulting strain (FS1Δ*IMP3*::*ADE2* + pCEN-*IMP3*) was transformed with the *IMP3Q* allele, which was amplified from pUC19-*IMPQ* with IMP_785_ and IMP_1924_. Ura^+^/Ade^-^ red transformants were selected and growth was analysed in absence of pCEN-*IMP3* on 5-FOA plates. A viable clone was verified by sequencing, resulting in the IMPQ strain.

**Polysome and ribosomal subunit analysis**

Cycloheximide 0.1 mg/ml was added to cultures immediately before harvesting. The cells were washed and broken in (10 mM Tris-HCl [pH 7.5], 100 mM NaCl, 30 mM MgCl2) in presence of cycloheximide. A total of 5 absorption units at 260 nm (200 µg of RNA) were loaded onto 11.4 ml of 15 to 50% linear sucrose gradients. Gradients were centrifuged at 39000 rpm in a Kontron SW41 rotor at 4°C for 3 hours, then analysed.

To determine the 40S and 60S ratio, after addition of cycloheximide 0.1 mg/ml to cultures, cells were washed and broken in low-Mg^2+^ buffer (50 mM Tris-HCl, pH 7.4; 50 mM NaCl; and 1 mM DTT). An amount of 5 A_260_ was then loaded on to a ~12 ml 10%-30% sucrose gradient and analysed as described for polysomes.

**Temperature and drug sensitivity assays**

The following drugs were assayed: G418 (10µg/mL; 25µg/mL and 50µg/mL), paromomycin (150µg/mL, 250µg/mL, 500µg/mL), ribostamycin (250µg/mL, 500µg/mL), tobramycin (250µg/mL, 500µg/mL), neomycin (0.2mg/mL, 1mg/mL), kanamycin (250µg/mL, 1mg/mL), bleomycin (250ng/L, 500ng/mL, 1µg/mL), phleomycin (1µg/L, 1.5µg/mL, 2µg/mL, 4µg/mL), camptothecin (0.5µg/mL, 1µg/mL, 10µg/mL, 20µg/mL), hydroxyurea (0.01M, 0.02M, 0.05M, 0.1M), methyl methanesulfonate (0.001%, 0.005%, 0.01%, 0.05%), and H_2_O_2_ (0.5µg/mL, 1µg/mL, 1,5µg/mL, 2µg/mL) (all Sigma purchased).
